# Comparison of the antibiotic resistance mechanisms in a gram-positive and a gram-negative bacterium by gene networks analysis

**DOI:** 10.1371/journal.pone.0311434

**Published:** 2024-11-15

**Authors:** Nafiseh Davati, Abozar Ghorbani

**Affiliations:** 1 Faculty of Food Industry, Department of Food Science and Technology, Bu-Ali Sina University, Hamedan, Iran; 2 Nuclear Science and Technology Research Institute (NSTRI), Nuclear Agriculture Research School, Karaj, Iran; Universidad Autonoma de Chihuahua, MEXICO

## Abstract

Nowadays, the emergence of some microbial species resistant to antibiotics, both gram-positive and gram-negative bacteria, is due to changes in molecular activities, biological processes and their cellular structure in order to survive. The aim of the gene network analysis for the drug-resistant *Enterococcus faecium* as gram-positive and *Salmonella Typhimurium* as gram-negative bacteria was to gain insights into the important interactions between hub genes involved in key molecular pathways associated with cellular adaptations and the comparison of survival mechanisms of these two bacteria exposed to ciprofloxacin. To identify the gene clusters and hub genes, the gene networks in drug-resistant *E*. *faecium* and *S*. *Typhimurium* were analyzed using Cytoscape. Subsequently, the putative regulatory elements were found by examining the promoter regions of the hub genes and their gene ontology (GO) was determined. In addition, the interaction between milRNAs and up-regulated genes was predicted. *RcsC* and *D920_01853* have been identified as the most important of the hub genes in *S*. *Typhimurium* and *E*. *faecium*, respectively. The enrichment analysis of hub genes revealed the importance of efflux pumps, and different enzymatic and binding activities in both bacteria. However, *E*. *faecium* specifically increases phospholipid biosynthesis and isopentenyl diphosphate biosynthesis, whereas *S*. *Typhimurium* focuses on phosphorelay signal transduction, transcriptional regulation, and protein autophosphorylation. The similarities in the GO findings of the promoters suggest common pathways for survival and basic physiological functions of both bacteria, including peptidoglycan production, glucose transport and cellular homeostasis. The genes with the most interactions with milRNAs include *dpiB*, *rcsC* and *kdpD* in *S*. *Typhimurium* and *EFAU004_01228*, *EFAU004_02016* and *EFAU004_00870* in *E*. *faecium*, respectively. The results showed that gram-positive and gram-negative bacteria have different mechanisms to survive under antibiotic stress. By deciphering their intricate adaptations, we can develop more effective therapeutic approaches and combat the challenges posed by multidrug-resistant bacteria.

## 1. Introduction

Fluoroquinolone antibiotics (FQs), as synthetic antibacterial drugs, are used to treat a variety of bacterial infections. However, improper usage of these drugs has increased bacteria’s resistance to FQ and microbial infections become difficult to treat [1]. Ciprofloxacin is an antibiotic that belongs to the fluoroquinolone (FQ) class and is used to treat both gram-positive and gram-negative bacteria [2,3]. ESKAPE is one of the most significant bacterial groups implicated in infections, including *Staphylococcus aureus*, *Enterococcus faecium*, *Acinetobacter baumannii*, *Enterobacter* spp., *Pseudomonas aeruginosa*, and *Klebsiella pneumoniae*, involved in infections and distinguished by its resistance to multidrug [4]. Recently, there have been several reports of ciprofloxacin resistance in *Salmonella Typhimurium*, *Bacillus anthracis*, *E*. *faecium*, *Enterococci* spp., *Neisseria gonorrhoeae*, *K*. *pneumoniae*, *P*. *aeruginosa*, and *Escherichia coli* [5–8]. DNA replication, repair, and recombination of bacteria are affected by FQs through inhibiting DNA gyrase (*gyrA*) enzymes [6]. Hence, the mutations in the *gyrA* gene or efflux pumps may be the cause of this drug resistance [9]. These antibiotic-resistant species can be transmitted to humans through food. Davati (2022) reported that the local cheeses could be responsible for transmitting a variety of antibiotic-resistant microbial species, special *Enterococcus* spp. [10]. In addition to the selection advantage derived from both innate and acquired antibiotic resistance features, *E*. *faecium*’s remarkable genomic flexibility and adaptable metabolism enable it to withstand a wide range of environmental stressors [11]. To prevent the development and spread of FQ resistance, it is crucial to understand the mechanisms underlying bacteria with increased drug resistance. Investigating gene networks can shed light on the relationships between genes and proteins linked to particular biochemical processes, which can help us comprehend the physiological state of the organism [12,13]. Following previous studies and in agreement with Li, Dai [8], who pointed out the need to perform gene network analysis of FQs-resistant *Salmonella* in further studies and also to compare the gene network in this FQs-resistant gram-negative bacteria with the gene network in an FQs-resistant gram-positive bacteria, the aim of this study was to investigate the gene interaction networks of the bacteria *E*. *faecium* (gram-positive) and *S*. *Typhimurium* (gram-negative) in order to identify key gene clusters and hub genes associated with their response to ciprofloxacin. Analysis of previously reported differentially expressed genes (DEGs) of these drug-resistant bacteria [7,8] revealed crucial molecular pathways and adaptations in *E*. *faecium* and *S*. *Typhimurium* when exposed to ciprofloxacin. Subsequently, the putative regulatory elements were found by examining the promoter regions of the hub genes and their gene ontology (GO) was determined. The survival mechanisms of these two bacteria were then compared. The results could contribute to the development of more effective therapeutic approaches and to combating the challenges posed by multidrug-resistant bacteria.

## 2. Materials and methods

### 2.1. Selection of DEGs

Data were selected from two appropriate articles [7,8] and obtained as follows. The gene expression data of the drug-resistant species of *E*. *faecium* and *S*. *Typhimurium* were downloaded from the supplementary data of https://doi.org/10.1128/aac.02763-16 [7] under Gene Expression Omnibus ID: GSE94507 and https://doi.org/10.1371/journal.pone.0175234 [8] under SRA ID: SRP100813. In this study, the up-regulated genes with fold change > 2 (p < 0.05) associated with drug-resistant mechanisms were evaluated for the comparison of drug-resistant *E*. *faecium* and drug-resistant *S*. *Typhimurium*. The count of retrieved up- and down-regulated genes found in drug-resistant species of *Salmonella Typhimurium* and *Enterococcus faecium* were listed in **S1 Table.**

### 2.2. The Retrieval of the protein-protein interaction (PPI) networks

To understand the interactions between the significantly up-regulated genes of *E*. *faecium* and *S*. *Typhimurium*, the PPI networks were created using the STRING database (version 12) (https://string-db.org/). *E*. *faecium* and *Salmonella enterica* subsp. *enterica* serovar *Typhimurium* were selected as reference organisms. The significant interactions with a medium confidence value of more than 0.4 were selected as significant interactions separately for *E*. *faecium* and *S*. *Typhimurium* [14].

### 2.3. PPIs networks analysis

The analysis of the PPI networks retrieved from the STRING database was performed with Cytoscape-v3.9.1. The CytoHubba plugin was used to identify the hub genes. The hub genes have the most interactions with other genes in the networks and play an important role in the gene network structures. The topological algorithms Maximum Neighborhood Component (MNC), Density of Maximum Neighborhood Component (DMNC), Maximal Clique Centrality (MCC), and Degree were used to determine the top genes in the PPI networks [15]. Cluster analysis of the PPI network was carried out using the Identifying Protein Complex Algorithm (IPCA) of the Cytocluster plugin as a density-based clustering algorithm to predict protein complex clusters [16]. The following settings were considered:

Tin threshold = 0.5, shortest path length = 2, Complex size threshold = 10.

IPCA is a density-based clustering algorithm that can detect dense subgraphs in PPI networks. Based on their connectivity values, the clusters were scored and the most significant protein complex clusters (rank 1–4) were found and displayed in the network.

### 2.4. Pathway enrichment and Gene Ontology (GO)

Pathway enrichment and GO analyses were carried out to gain insights into the molecular functions and biological processes related to the hub genes and their connections. The STRING database was used to perform pathway enrichment analysis on the networks containing the hub genes and their interactions. The enriched pathways and biological processes that are statistically overrepresented among the up-regulated hub genes can be identified by the pathway enrichment analysis. The results of the pathway enrichment analysis provide important insights into the functional significance of hub genes in the context of antibiotic resistance. The hub genes were categorized according to cellular components (CC), molecular functions (MF), and biological processes (BP) using GO analysis [17]. The search for the functional annotations and features of these antibiotic-resistance genes is enabled by assigning GO keywords to the hub genes. Based on the annotations derived from GO analysis, the genes can be categorized into different functional groups, which provides important information about the functions and interactions of individual genes within cellular processes. The cellular components, biological processes, and molecular functions that are especially relevant in the context of antibiotic resistance are highlighted by the GO keywords associated with the hub genes.

### 2.5. Promoter analysis of Hub genes

We aimed to uncover putative regulatory elements (cis-elements) involved in the response to antibiotic treatment by examining the promoter regions of the differentially expressed hub genes. The 200 kbp upstream flanking regions of the hub up-regulated genes as promoter sequences were obtained from NCBI. The database of prokaryote DNA for the bacterial TF motif with threshold E-value < 10 was used to identify the known motifs using the motif comparison tool (Tomtom) version 5.5.5 (https://meme-suite.org/meme/tools/tomtom) [18]. GO for motifs (GOMo) version 5.5.5 was then used to determine the potential roles of the detected motifs (https://meme-suite.org/meme/tools/gomo) [19].

### 2.6. Prediction of up-regulated genes interaction with miRNAs

Data on small RNAs, including microRNA-like RNA fragments (miRNA), are from https://www.mirbase.org. The interaction between miRNAs and up-regulated genes of *S*. *Typhimurium* and *E*. *faecium* was predicted using the analysis server psRNATarget (2017 Update) (http://www/.zhaolab.org/psRNATarget/) by entering genes as targets and miRNAs as queries. In general, the following settings were considered: Number of top targets: 200, the penalty for G:U pair: 0.5, extra weight in the seed region: 1.5, number of allowed mismatches in the seed region: 2, the penalty for the opening gap: 2, expectation: 5, the penalty for other mismatches: 1, seed region: 2–13 NT, HSP size: 19, the penalty for extending gap: 0.5, and translation inhibition range: 10 NT-11 NT [20]. Interaction networks of miRNAs with genes and were created using Cytoscape (version 3.9.1). The nodes in this network consisted of miRNAs, while the genes acted as targets of the miRNAs. The CytoHubba plugin was used to identify the genes that have the most interactions with miRNAs in the *S*. *Typhimurium* and *E*. *faecium* networks using Degree’s topological algorithm.

## 3. Results

Ciprofloxacin has been used for about thirty years to treat a variety of illnesses, such as urinary tract infections, endocarditis, lower respiratory tract infections, gastrointestinal tract infections, and skin, and soft tissue infections. The main mechanism of action of ciprofloxacin is to prevent DNA replication by blocking the A subunit of DNA gyrase and exerting additional influence on the components of cell walls. A rise in ciprofloxacin resistance has been observed in several bacteria both gram-positive and gram-negative bacteria over time, despite the antibiotic’s exceptional properties. In the present study, gene clusters and hub genes in *E*. *faecium* and *S*. *Typhimurium* that might be involved in cellular adaptions in response to inhibitory or subinhibitory concentrations of ciprofloxacin were found using network analysis.

### 3.1. Gene network analysis

Earlier studies by Sinel, Cacaci [7] and Li, Dai [8] investigated the mechanism of ciprofloxacin resistance based on the gene expression patterns of the resistant species *E*. *faecium* and *S*. *Typhimurium*, respectively. Nevertheless, there might be other unknown genes that are crucial for this stress response and that are part of the gene network activated by ciprofloxacin resistance. Therefore, a network analysis was performed to find other important genes associated with inhibitory or sub-inhibitory levels of ciprofloxacin. The possible protein-protein interactions between DEGs were visualized using the STRING database. 23 genes with 104 interactions for *S*. *Typhimurium* and 28 genes with 237 interactions for *E*. *faecium* were identified using the interaction data from this database. The topological parameters (**S2 Table**) for the analysis of the subnetworks of upregulated genes and their interactions in the drug-resistant species of *S*. *Typhimurium* and *E*. *faecium* are accessible.

### 3.2. Identification of Hub genes

The list of the top of hub up-regulated genes found in the network analysis of *S*. *Typhimurium* and *E*. *faecium* can be found in **S3 Table**. Hub genes, which have the most gene interactions, can involved as important players in many different biological processes and molecular activities. These hub genes include *rcsC*, *narL*, *phoQ*, *basR*, *phoP*, *rstB*, *envZ*, and *rstA* in *S*. *Typhimurium* and include *D920_01853*, *D920_01857*, *D920_02619*, *D920_01002*, *D920_02133*, *D920_02981*, *D920_00703*, *D920_02792*, *D920_02266*, and *D920_03079* in *E*. *faecium*.

### 3.3. GO analysis

**Fig 1:**
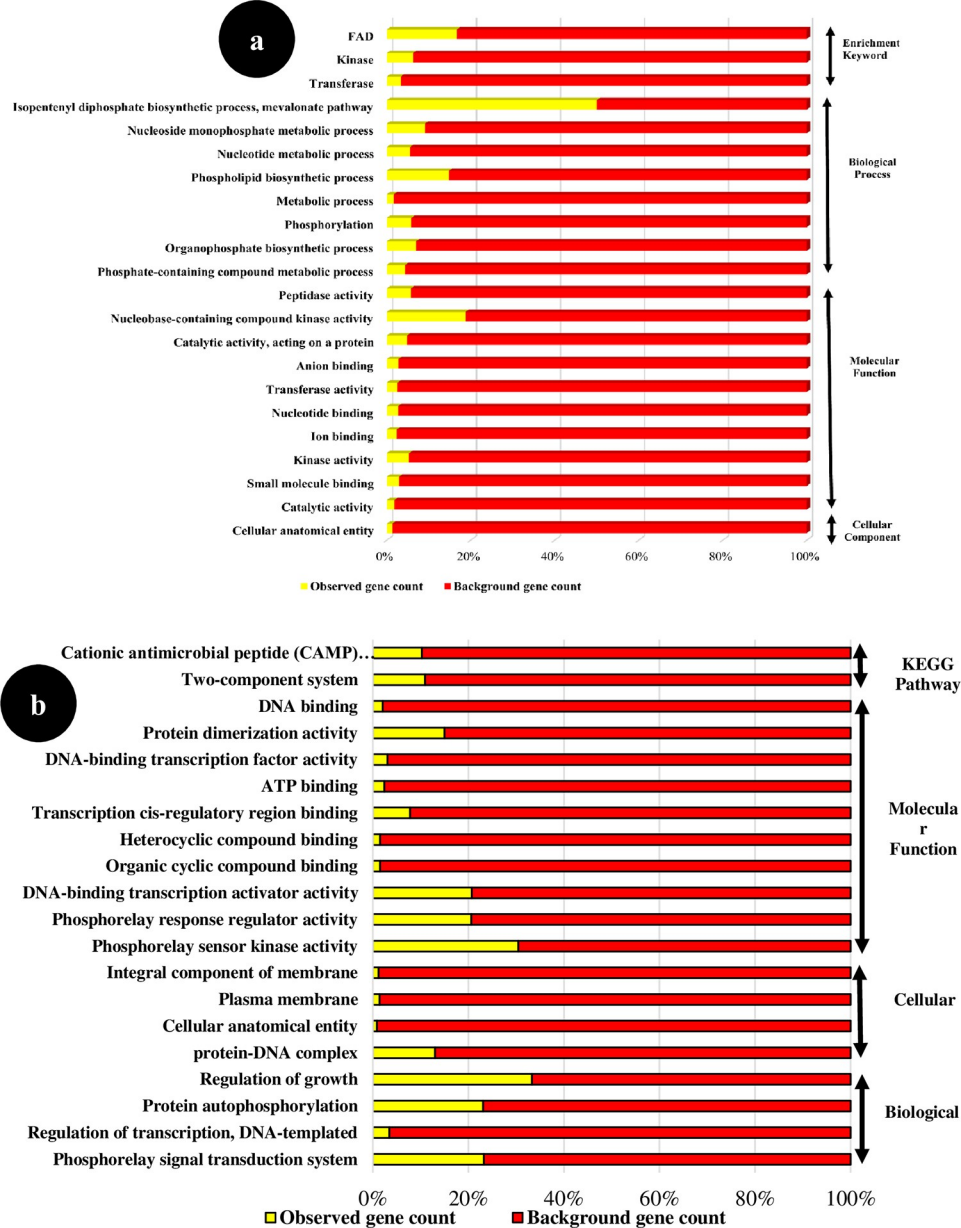
Gene Ontology enrichment analysis of hub genes of *Enterococcus faecium* (a) and *Salmonella Typhimurium* (b).

To learn more about the biological mechanisms, molecular roles, and cellular components associated with the hub genes found in *E*. *faecium* and *S*. *Typhimurium* that are affected by inhibitory or subinhibitory concentrations of ciprofloxacin, an enrichment analysis of the functional genes was performed. **Fig 1** shows a summary of the results. Numerous significant functions connected to the hub genes involved in BP, MF, and CC were revealed by the functional enrichment analysis for *E*. *faecium* and *S*. *Typhimurium*.

### 3.4. Clustering analysis

**Fig 2:**
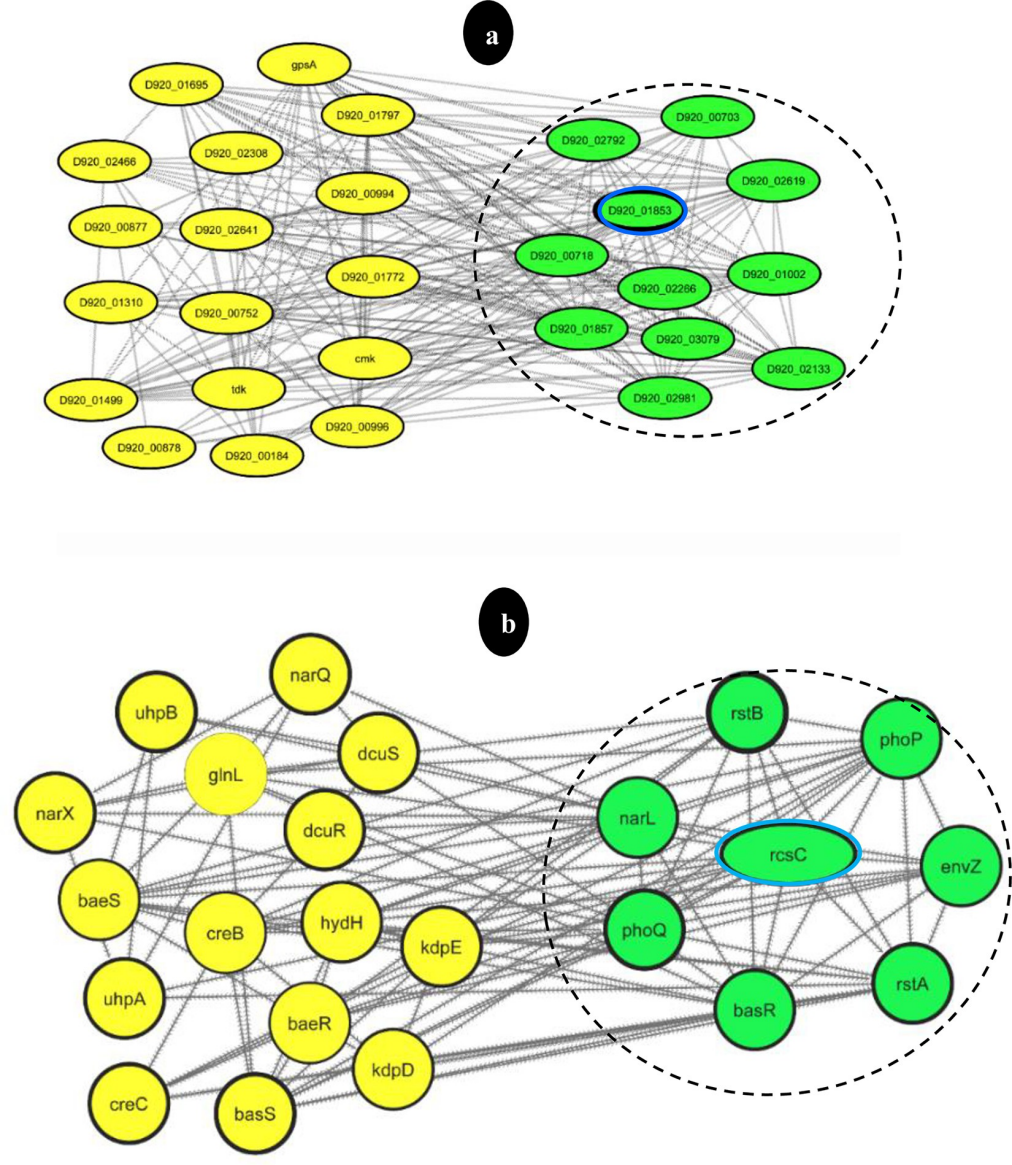
The gene network of *Enterococcus faecium* (a) and *Salmonella Typhimurium* (b). Hub genes are shown in green and the major hub gene is visualized within a blue ring. FC amounts of *S*. *Typhimurium* are visualized based on the thickness of the knot border. The rank 1 clusters in gene networks are visualized within a black dashed ring.

In protein-protein interaction networks, the IPCA algorithm helps to determine which protein complex clusters are most important. The clusters were ranked according to their connectivity values using IPCA analysis. As can be seen in **Table 1**, this algorithm determines the weight of each edge by finding the common neighbor of two connected nodes [16,21]. The protein complex clusters with the highest scores and significance were represented by the rank 1 clusters. In **Fig 2**, only the rank 1 cluster (shown within a black ring) was shown in *E*. *faecium* and *S*. *Typhimurium*. Cluster analysis can be used to find groups of genes that show coordinated expression patterns and are likely to be involved in related biological processes or pathways. This research sheds light on the functional organization and possible interactions between different elements of the cellular response to ciprofloxacin in *S*. *Typhimurium* and *E*. *faecium* by clustering the hub genes.

**Table 1:** Cluster analysis of PPI networks of drug-resistant species of *Enterococcus faecium* and *Salmonella Typhimurium* using IPCA.

***S*. *Typhimurium***	**Nodes**	**Edges**	**Rank**	**KEGG Pathways** **(or Annotated Keywords)**
	14	64	1	Two-component system Cationic antimicrobial peptide resistance
	11	43	2	
	10	34	3	
	8	19	4	Two-component system
*E*. *faecium*	22	187	1	Pyrimidine metabolism (Transferase, Kinase, FAD)
	22	190	2	
	22	191	3	
	21	177	4	

### 3.5. Promoter motif analysis

We subjected the 200 bp upstream flanking regions of the hub DEGs in *E*. *faecium* and *S*. *Typhimurium* to promoter motif analysis to gain a better understanding of the regulatory mechanisms underlying the differential expression of hub genes in response to ciprofloxacin stress. This analysis aimed to find conserved motifs that may be associated with the regulation of these genes. Based on the prokaryote DNA motif database for bacterial transcription factor motifs, we searched the promoter sequences of *E*. *faecium* and *S*. *Typhimurium* for known motifs using the MEME motif comparison tool (Tomtom). We used an E-value of less than 10 to identify meaningful matches. In addition, we determined the likely functions of the discovered motifs using the GO for motifs (GOMo) program. The analysis revealed the presence of several motifs with known GO annotations in the promoter regions of the hub genes (**Tables 2 and 3**). These motifs ranged in length from 11 to 48 base pairs. Understanding the biological and molecular roles associated with the motifs found was enabled by this analysis. The results also showed that there were similar GO results of motifs between *E*. *faecium* as a gram-positive bacteria and *S*. *Typhimurium* as a gram-negative bacteria, but these two bacteria also have some differences. Several motifs in both *E*. *faecium* and *S*. *Typhimurium* were found to be associated with biological processes such as cellular homeostasis, cellular carbohydrate catabolic process, peptidoglycan-based cell wall organization, serine family amino acid catabolic process, carbohydrate transport, cytochrome complex assembly, peptidoglycan biosynthetic process, and proton transport. However, motifs with biological processes associated with biofilm formation, biological regulation, and multi-organism processes were only discovered in *E*. *faecium*. In addition, motifs with biological processes related to aerobic respiration, alcohol catabolic process, chemical homeostasis, macromolecule catabolic process, monosaccharide catabolic process, pentose catabolic process, transcription, DNA-dependent, and pentose metabolic process were only found in *S*. *Typhimurium*. Motifs with different GO are associated with molecular functions, such as driven active transmembrane transporter activity, heme binding, intramolecular oxidoreductase activity, racemase and epimerase activity, sugar transmembrane transporter activity, active transmembrane transporter activity, oxidoreduction, interconverting aldoses and ketoses, and acting on carbohydrates and derivatives in both *E*. *faecium* and *S*. *Typhimurium*. But motifs with molecular functions associated with cation transmembrane transporter activity, oligopeptide, ATPase activity, ion transmembrane transporter activity, transporting ATPase activity, and coupled to transmembrane movement of substances were only discovered in *E*. *faecium*. In addition, motifs with molecular functions associated with electron carrier activity, glutamate decarboxylase, mismatched DNA binding, and zinc ion binding activity were only discovered in S. *Typhimurium*. Interestingly, a GO motif associated with cellular components was only discovered in *E*. *faecium*. This motif (motif 6) was associated with cellular components of the ATP-binding cassette (ABC) transporter complex. These results suggest that the motifs found in the hub DEG promoter regions may be important regulators of gene expression when exposed to ciprofloxacin. Functional studies and additional experimental validation are required to confirm the regulatory relationships between these motifs and their associated genes. Nonetheless, the study of promoter motifs provides important insights into putative regulatory mechanisms supporting the differential expression of hub genes upon ciprofloxacin stress. In general, the study of promoter motifs provides an additional level of understanding of the changes in gene expression associated with antibiotic resistance. The complex network of relationships that determines the cellular adaptations of *S*. *Typhimurium* and *E*. *faecium* during ciprofloxacin resistance can be partially elucidated by identifying conserved regulatory motifs.

**Table 2:** The conserved motifs in promoter of hub up-regulated genes of *Enterococcus faecium* by MEME analysis.

Hub DEGs	Motif NO.	E-value	Width	Best match in Prokaryote DNA	GO term identified by GOMO
***EFAU004_00167***	Motif 1	3.57e+00	15	EXPREG_000016a0	**BP** proton transport- cellular macromolecule catabolic process- cellular carbohydrate catabolic process **MF** oxidoreduction-driven active transmembrane transporter activity- racemase and epimerase activity, acting on carbohydrates and derivatives
***EFAU004_00538***	Motif 2	2.31e+00	41	EXPREG_00000340	**BP** cytochrome complex assembly- serine family amino acid catabolic process- biofilm formation **MF** active transmembrane transporter activity
	Motif 3	3.14e+00	29	EXPREG_00000ff0	**MF** active transmembrane transporter activity- heme binding- intramolecular oxidoreductase activity, interconverting aldoses and ketoses **BP** peptidoglycan biosynthetic process- peptidoglycan-based cell wall organization
***EFAU004_00900***	Motif 4	1.10e+00	47	EXPREG_00000340	**BP** cytochrome complex assembly- serine family amino acid catabolic process- biofilm formation **MF** active transmembrane transporter activity
	Motif 5	1.97e-01	27	EXPREG_000008e0	**MF** active transmembrane transporter activity- ion transmembrane transporter activity
***EFAU004_01228***	Motif 6	5.66e-01	11	EXPREG_000010c0	**MF** cation transmembrane transporter activity- ATPase activity, coupled to transmembrane movement of substances- sugar transmembrane transporter activity **BP** carbohydrate transport **CC** ATP-binding cassette (ABC) transporter complex
***EFAU004_01710***	Motif 7	3.42e+00	48	EXPREG_00000340	**BP** cytochrome complex assembly- multi-organism process- serine family amino acid catabolic process
***EFAU004_02225***	Motif 8	3.71e+00	15	EXPREG_000001b0	**MF** active transmembrane transporter activity- cation transmembrane transporter activity **BP** transport
	Motif 9	2.87e-02	45	EXPREG_00000340	**BP** cytochrome complex assembly- multi-organism process- serine family amino acid catabolic process
***EFAU004_02486***	Motif 10	4.78e-01	21	EXPREG_00000050	**MF** ion transmembrane transporter activity- heme binding- oligopeptide-transporting ATPase activity **BP** transport- cellular homeostasis
	Motif 11	1.99e+00	25	EXPREG_00000360	**BP** biological regulation
	Motif 12	7.14e-02	34	EXPREG_00000340	**BP** cytochrome complex assembly-multi-organism process- serine family amino acid catabolic process

**BP:** Biological process **MF:** Molecular function **CC:** Cellular Component.

**Table 3:** The conserved motifs in promoter of hub up-regulated genes of *Salmonella Typhimurium* by MEME analysis.

Hub DEGs	Motif NO.	E-value	Width	Best match in Prokaryote DNA	GO term identified by GOMO
***rstB***	Motif 1	3.84e+00	43	EXPREG_00000470	**BP** pentose metabolic process, alcohol catabolic process
***rstA***	Motif 2	4.15e+00	21	EXPREG_00000850	**BP** carbohydrate transport- pentose catabolic process, macromolecule catabolic process **MF** sugar transmembrane transporter activity, intramolecular oxidoreductase activity, interconverting aldoses and ketoses
	Motif 3	5.91e+00	19	EXPREG_00001000	**BP** serine family amino acid catabolic process
	Motif 4	9.77e+00	22	EXPREG_00001030	**BP** cellular carbohydrate catabolic process, cellular macromolecule catabolic process **MF** intramolecular oxidoreductase activity, interconverting aldoses and ketose
***rcsC***	Motif 5	8.73e+00	17	EXPREG_00000850	**BP** carbohydrate transport- pentose catabolic process, macromolecule catabolic process **MF** sugar transmembrane transporter activity, intramolecular oxidoreductase activity, interconverting aldoses and ketoses
	Motif 6	9.39e+00	20	EXPREG_00001000	**BP** serine family amino acid catabolic process
***phoQ***	Motif 7	9.94e-01	42	EXPREG_00000470	**BP** pentose catabolic process
	Motif 8	7.77e+00	27	EXPREG_00001440	**MF** electron carrier activity **BP** cytochrome complex assembly, aerobic respiration
***narL***	Motif 9	4.72e+00	17	EXPREG_000012b0	**MF** zinc ion binding- glutamate decarboxylase activity, mismatched DNA binding, oxidoreduction-driven active transmembrane transporter activity **BP** transcription, DNA-dependent
***envZ***	Motif 10	2.09e+00	26	EXPREG_00000f90	**BP** cellular carbohydrate catabolic process, cellular macromolecule catabolic process
	Motif 11	3.35e+00	32	EXPREG_00000ff0	**MF** active transmembrane transporter activity- heme binding **BP** peptidoglycan biosynthetic process- monosaccharide catabolic process- peptidoglycan-based cell wall organization
***basR***	Motif 12	7.41e+00	15	EXPREG_000016a0	**BP** proton transport, cellular macromolecule catabolic process, cellular carbohydrate catabolic process **MF** racemase and epimerase activity, acting on carbohydrates and derivatives, oxidoreduction-driven active transmembrane transporter activity
	Motif 13	4.03e+00	17	EXPREG_00000b00	**BP** cellular-homeostasis, chemical homeostasis

**BP:** Biological process **MF:** Molecular function.

### 3.6. Predicted interactions between up-regulated genes and milRNAs

**Fig 3:**
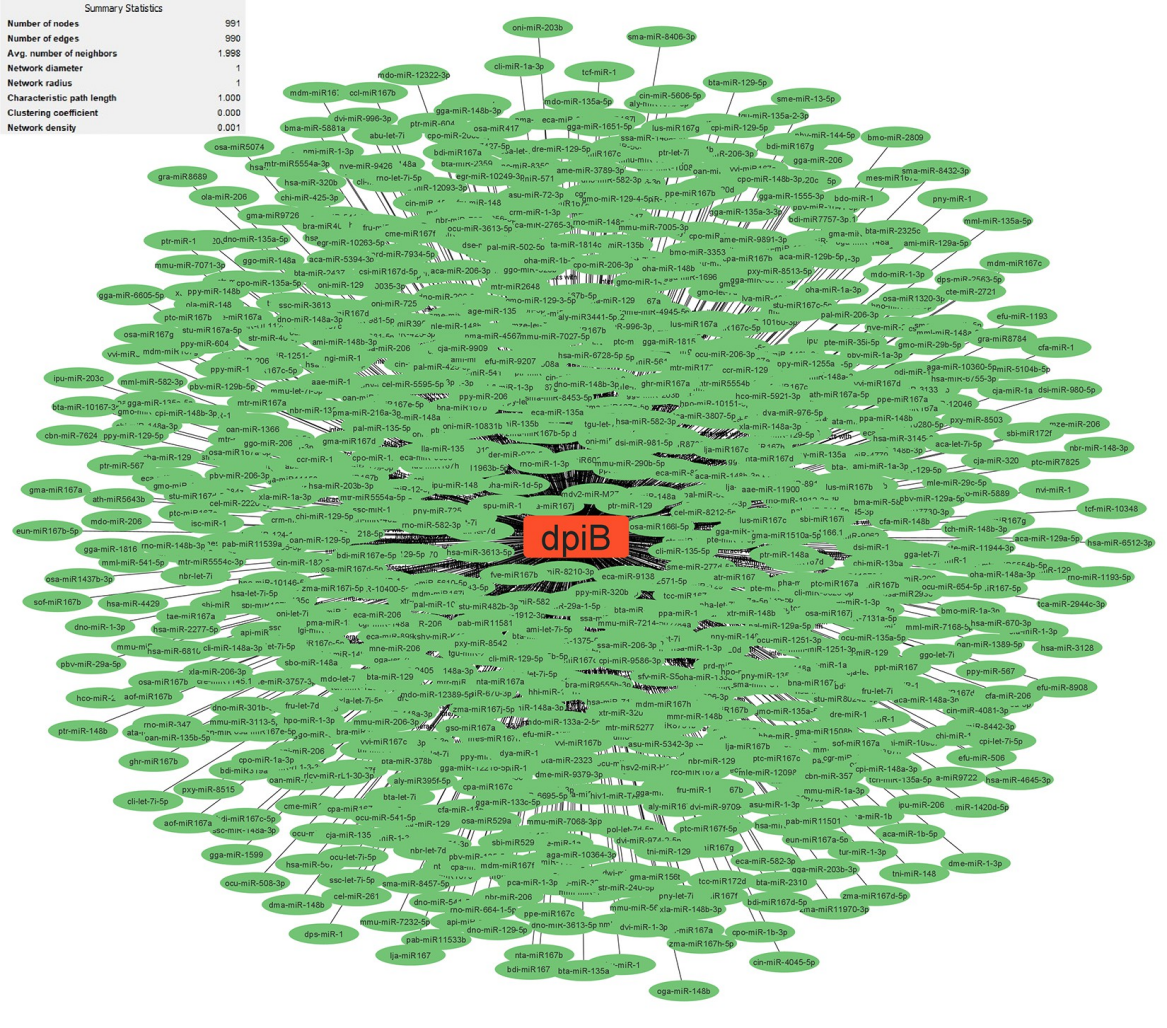
The network of predicted relationships between *dpiB* gene (red square) and milRNAs (green oval) in *Salmonella Typhimurium*.

**Fig 4:**
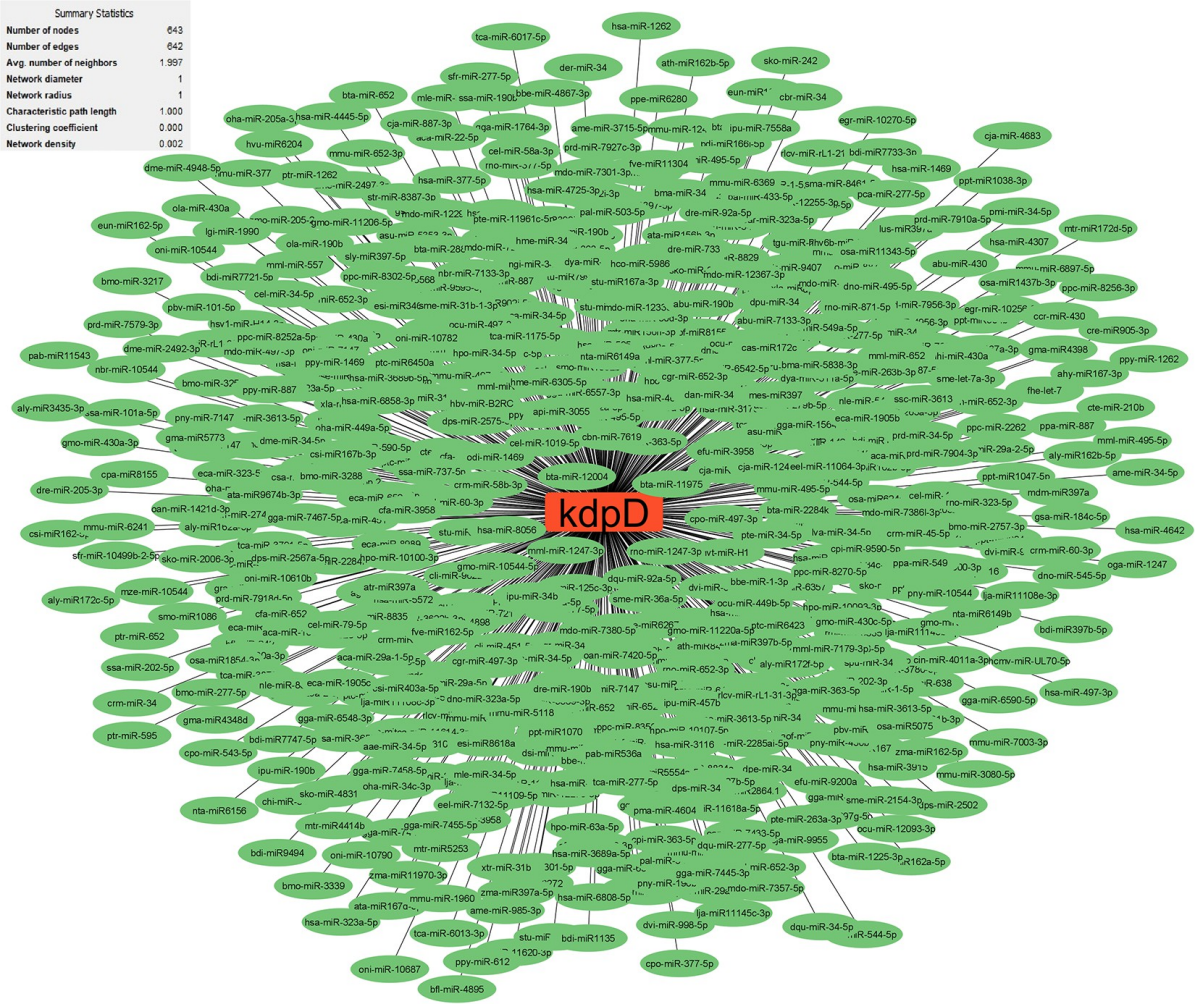
The network of predicted relationships between *kdpD* gene (red square) and milRNAs (green oval) in *Salmonella Typhimurium*.

**Fig 5:**
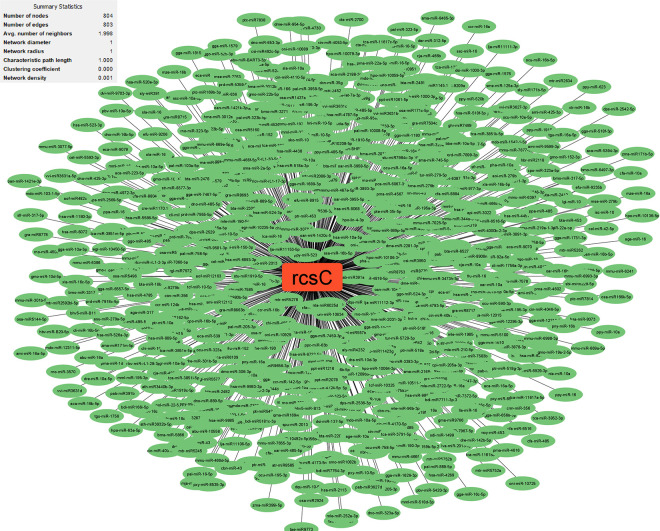
The network of predicted relationships between *rcsC* gene (red square) and milRNAs (green oval) in *Salmonella Typhimurium*.

**Fig 6:**
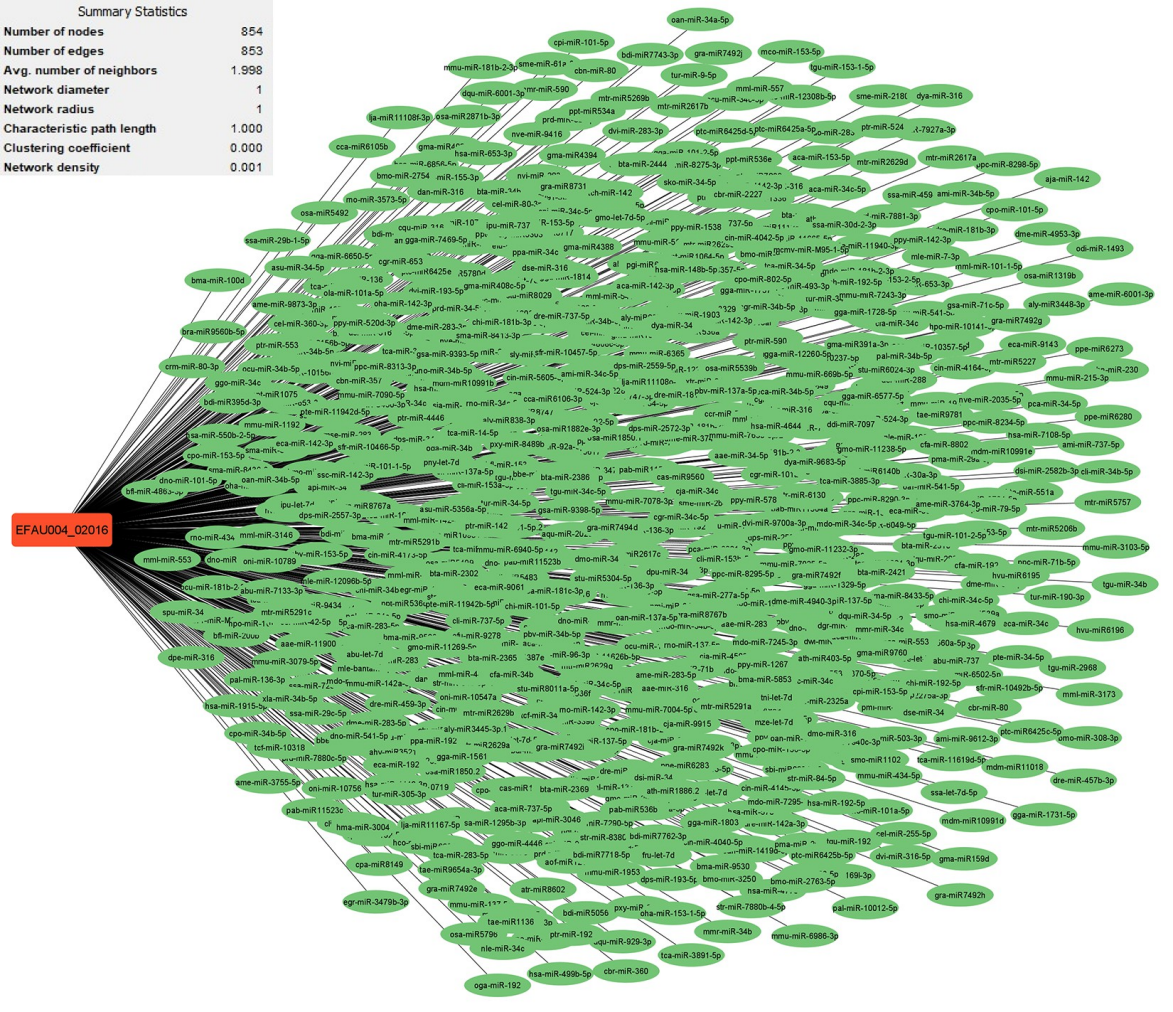
The network of predicted relationships between *EFAU004_02016* gene (red square) and milRNAs (green oval) in *Enterococcus faecium*.

**Fig 7:**
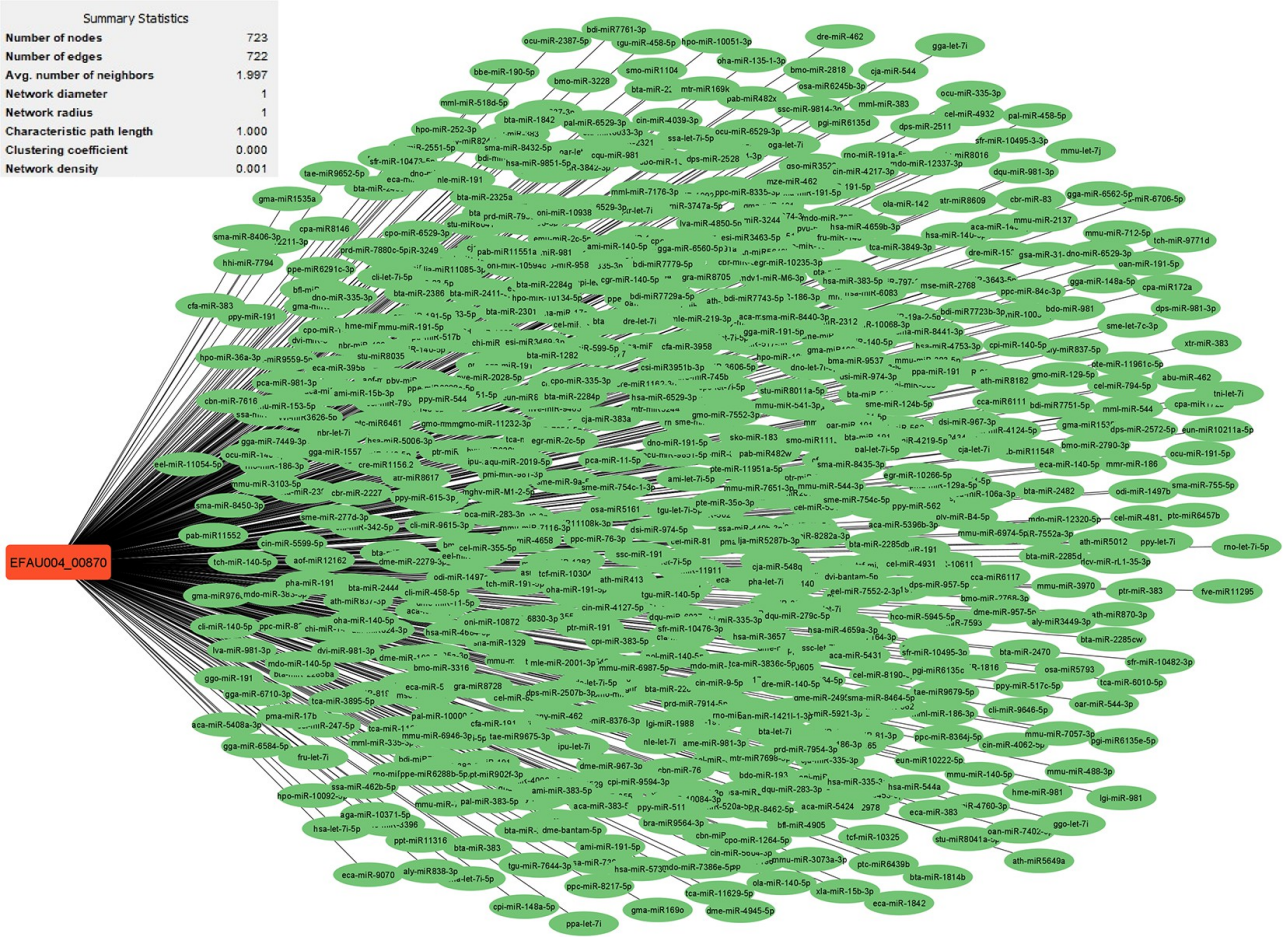
The network of predicted relationships between *EFAU004_00870* gene (red square) and milRNAs (green oval) in *Enterococcus faecium*.

**Fig 8:**
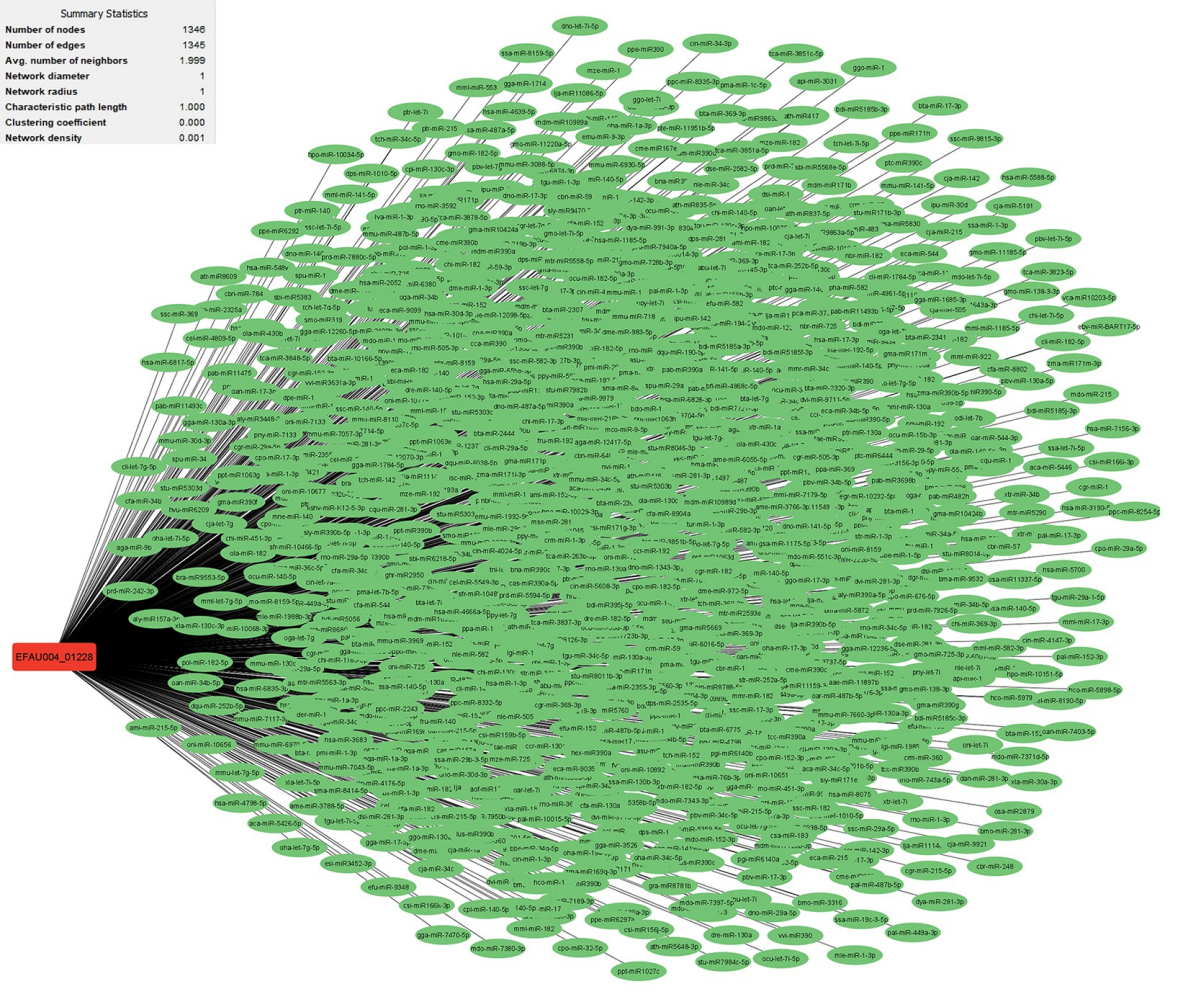
The network of predicted relationships between *EFAU004_01228* gene (red square) and milRNAs (green oval) in *Enterococcus faecium*.

Research has uncovered the role of milRNAs in *Salmonella* infections, particularly in how they help the bacteria express their virulence genes. These milRNAs are crucial for regulating gene expression in bacterial cells and influence functions such as virulence and environmental responses. For example, a specific microRNA-like RNA fragment called Sal-1 is produced by *Salmonella* in infected cells to facilitate intracellular survival. This demonstrates the sophisticated mechanisms that bacteria such as *Salmonella* use to adapt to and survive in their host environment [22]. The results showed that the genes with the most interactions with milRNAs in *S*. *Typhimurium* (**Figs 3–5**) include *dpiB* (990 interactions), *rcsC* (803 interactions) and *kdpD* (642 interactions), respectively, and in *E*. *faecium* (**Figs 6–8**) *EFAU004_01228* (1345 interactions), *EFAU004_02016* (853 interactions) and *EFAU004_00870* (722 interactions), respectively (for more detailed information please see **S1 File**). The prediction results of interactions between upregulated genes and milRNAs showed that even the up-regulated genes of *dpiB* and *kdpD* as non-hub genes can be effective for cellular survival through interactions with milRNAs under antibiotic stress. Based on the string database, *rcsC*, *dpiB* and *kdpD* are associated with the two-component system sensory histidine kinase in two-component regulatory systems. Consequently, most milRNAs could be involved in the regulation of gene expression of two-component systems affected by inhibitory or sub-inhibitory antibiotic concentrations in negative Gram bacteria such as *Salmonella*. In a Gram-positive bacterium such as Enterococcus, most interactions of milRNAs were dedicated to genes associated with transferase activity (*EFAU004_02016*) and antibiotic binding protein (*EFAU004_00870*). The antibiotic binding protein and also the transferase by altering the molecules of antibiotics contribute to antibiotic resistance, thereby reducing the effectiveness of the drugs.

## 4. Discussion

In our study, we investigated differentially expressed genes (DEGs) under both subinhibitory and inhibitory conditions to gain a comprehensive understanding of the mechanisms of antibiotic resistance in gram-positive and gram-negative bacteria. Despite originating from different experimental environments, the analysis of these datasets is justified because antibiotic resistance often involves overlapping pathways and mechanisms that may be affected by different levels of antibiotic exposure. By comparing the DEGs of both conditions, we aim to highlight common and non-common genes and resistance pathways while capturing a broader spectrum of bacterial responses. This approach improves our ability to identify novel resistance-related genes and contributes to a more comprehensive understanding of how bacteria adapt to different antibiotic exposures, enriching the insights from our comparative analysis.

### 4.1. Identification of Hub genes and GO analysis

The discovery of hub genes in gram-negative bacteria such as antibiotic-resistant *S*. *Typhimurium* and gram-positive bacteria such as antibiotic-resistant *E*. *faecium* reveals a fascinating aspect of the bacterial exposure to antibiotics. The activation of metabolic pathways in *S*. *Typhimurium* and *E*. *faecium* cells under ciprofloxacin resistance may have led to an increase in the expression of these genes. A previous study by Li, Dai [8] highlighted the importance of two-component regulatory systems in *S*. *Typhimurium* under the influence of ciprofloxacin. By altering gene expression and cellular processes, these systems play a crucial role in the adaptability of bacteria to environmental changes, including antibiotic stress [23]. Genes associated with the two-component regulatory system in *S*. *Typhimurium* include the *RcsB*, *RstA*, *NarX*, *PhoP*, *BasS*, and *PhoQ* genes as well as transcriptional regulatory proteins [8,24]. Therefore, the hub genes of *S*. *Typhimurium* associated with the sensory histidine kinases in the two-component regulatory system with *RcsB* and *RstA*, the response regulator in the two-component regulatory system with *NarX*, the sensory kinase protein in the two-component regulatory system with *PhoP*, the response regulators in the two-component regulatory system with *BasS* and *PhoQ*, and the transcriptional regulatory proteins were expected to be identified in this study. Among the hub genes of *S*. *Typhimurium*, the *rcsC* gene (sensory histidine kinases in the two-component regulatory system with *RcsB*) and *narL* gene (response regulator in the two-component regulatory system with *NarX*) had the highest significance based on the Hubba node method and their scores. In contrast, the hub genes in *E*. *faecium* were associated with the HAD superfamily hydrolase; acyltransferase; riboflavin biosynthetic protein; guanylate kinase; pyridine nucleotide disulfide; oxidoreductase; transglycosylase; ATPase; putative penicillin-binding protein; putative cysteine desulfurase; thioredoxin reductase. Among the identified hub genes of *E*. *faecium*, gene *D920_01853* (efc: *EFAU004_02486*) had the highest significance based on the method and its score. These hub genes are involved in several cellular processes, including metabolism, cell wall synthesis, and redox reactions, which may be critical for the survival and virulence of *E*. *faecium* under antibiotic stress. Similarly, the earlier study by Sinel, Cacaci [7] indicated the importance of HAD superfamily hydrolase, oxidoreductase, guanylate kinase, pyridine nucleotide disulfide ATPase, thioredoxin reductase, and nucleotide biosynthesis in *E*. *faecium*, which is affected by ciprofloxacin. The difference in hub genes between these two types of bacteria can be attributed to their different cell structures and the mechanisms they have evolved to cope with environmental stresses. Gram-negative bacteria such as *S*. *Typhimurium* have an outer membrane and a more complex cell envelope containing lipopolysaccharides, and they often rely on two-component systems to recognize and respond to external stimuli [25–27]. Gram-positive bacteria such as *E*. *faecium* do not have an outer membrane, but a thicker peptidoglycan layer. Their stress response may involve a broader range of metabolic adaptations and enzyme activities to maintain cellular function and integrity. Understanding these differences is critical for the development of targeted strategies to combat antibiotic resistance in these bacteria, as the key regulatory systems and metabolic pathways they rely on under stress are potential targets for new antimicrobial agents. The biological processes related to phosphate-containing compounds; phosphorylation; phospholipid biosynthetic process; nucleotide and nucleoside monophosphate metabolic process; and mevalonate pathway in antibiotic-resistant bacteria such as *Enterococcus* spp. may increase in response to inhibitory concentrations of ciprofloxacin for a variety of reasons. The increase in metabolic processes of phosphate-containing compounds may be related to the need to repair damaged DNA and membranes and to generate ATP to power efflux pumps that eject antibiotics from the cell. The increase in the organophosphate biosynthesis and phosphorylation process could be attributed to the synthesizing of the necessary intermediates and modifying the proteins to adapt to the stress induced by ciprofloxacin [28,29]. To maintain the integrity of the membrane and create new components for cell growth and division, the cell can improve its metabolic and phospholipid biosynthesis processes under the inhibitory concentration of ciprofloxacin [30]. To ensure a constant supply of nucleotides for DNA repair and replication in stress situations, the activities of nucleotide and nucleoside monophosphate metabolism are probably increased [31]. The production of isoprenoids, which are essential for the biosynthesis of cell walls and the modification of certain proteins, depends on isopentenyl diphosphate biosynthesis, which is a step in the mevalonate pathway. Overexpression of this system may be a response to the need for protein modification as a defense mechanism and for cell wall repair under ciprofloxacin stress [32,33]. The biological processes associated with the phosphorelay signal transduction system, transcriptional regulation, DNA templating, protein autophosphorylation, and growth regulation in antibiotic-resistant bacteria such as *Salmonella* spp. may increase in response to inhibitory concentrations of ciprofloxacin for a variety of reasons (see below). Transcriptional control of genes required for bacterial survival under environmental stresses, including antibiotic resistance, is one of the most important processes contributing to the development of antibiotic resistance. By controlling the expression of functional genes, transcriptional regulators (TRs) are essential for this process. A two-component system that enables bacteria to recognize and respond to changes in the environment, such as the presence of antibiotics, is the phosphorelay signal transduction system. This system consists of a response regulator (RR), which binds to DNA and alters gene expression when phosphorylated, and a histidine kinase, which autophosphorylates in response to an external stimulus. The RR, which typically binds to DNA and mediates a cellular response, receives information about the stimulus from a histidine kinase (HK). The response usually involves the phosphorylated response regulator binding to DNA to affect gene expression, which can lead to greater antibiotic resistance [29,34–36]. Several transcriptional regulators can be activated in response to inhibitory doses of the quinolone antibiotic ciprofloxacin. For example, drug-induced growth restriction in *Pseudomonas aeruginosa* activates transcriptional regulators such as the superoxide sensor regulator (SoxR) and the quorum sensing receptor (QscR) [30,37,38]. Furthermore, stimulation of efflux pump systems and other processes that support bacterial resistance to the antibiotic may occur in response to ciprofloxacin [29]. In addition, TRs bind to DNA to control the transcription of genes in a process known as DNA-templated transcription regulation. Mutations in TRs or their overexpression can lead to increased resistance to antibiotics by promoting the expression of efflux pump genes and other resistance mechanisms [39]. Protein autophosphorylation is a modification that can alter the function of proteins, including those involved in antibiotic resistance. Thus, phosphorylation of Ser/Thr/Tyr is a regulatory mechanism in bacteria that influences a range of activities, such as antibiotic resistance and pathogenicity [40]. This adaptation may involve changes in cell membrane composition, efflux pump activity, and other cellular processes that contribute to survival in the presence of the antibiotic. The increase in cellular components associated with the cellular anatomical entity in antibiotic-resistant *E*. *faecium* in response to the subinhibitory concentration of ciprofloxacin can be explained by several factors. As early mentioned, ciprofloxacin is a fluoroquinolone antibiotic that targets bacterial DNA gyrase and topoisomerase IV, which are crucial for DNA replication and repair. When bacteria are exposed to ciprofloxacin, it causes DNA damage and triggers a stress response that can lead to upregulation of genes involved in DNA repair, stress response, and efflux pump systems [28]. One of the most important defense mechanisms with which bacteria defend themselves against the effects of antibiotics is the efflux pump. Exposure to antibiotics can lead to increased expression of efflux pump genes, which increases efflux activity and lowers intracellular antibiotic concentrations [28,29,41]. In addition, bacteria can use the formation of biofilms as a defense mechanism in response to antibiotic stress. Bacteria form complex communities known as biofilms when they adhere to surfaces and become embedded in an extracellular matrix that they produce themselves. This matrix can be resistant to antibiotics, and bacteria living in biofilms can develop transient phenotypic resistance to them. Antibiotic tolerance can also be influenced by persister cells, i.e. dormant forms of normal bacterial cells found in biofilms [29]. In addition, activation of DNA damage triggers the SOS response, a global regulatory system that can trigger mutagenesis and DNA repair mechanisms, potentially leading to the emergence of antibiotic resistance [29]. For gram-negative bacteria such as *S*. *Typhimurium*, which has both innate and acquired resistance mechanisms, the plasma membrane and its essential components are of critical importance [28,29,41]. Reduced sensitivity to ciprofloxacin can also be due to changes in lipopolysaccharides (LPS) in the outer membrane of gram-negative bacteria such as *Salmonella* and membrane proteins [42]. In addition to these mechanisms, bacteria may also undergo genetic changes that lead to resistance, such as changes in the target enzymes DNA gyrase and topoisomerase IV, which reduce their susceptibility to inhibition by ciprofloxacin [43]. In addition, plasmids containing resistance genes, such as those expressing *Qnr* proteins, can increase an individual’s resistance to quinolones, such as ciprofloxacin. These plasmids can spread resistance within microbial communities by being transferred between bacteria [44]. In summary, the upregulation of genes related to the cellular components of *S*. *Typhimurium* in response to ciprofloxacin is a complex process involving multiple resistance mechanisms, including activation of efflux pumps, membrane modifications, DNA repair and genetic mutations. The drug-resistant bacteria have evolved multiple strategies to counteract the effects of antibiotics, and the upregulation of these molecular functions is part of their adaptive response to survive in the presence of the antibiotic. The biosynthesis of enzymes that render an antibiotic inactive is one of the most important resistance mechanisms. For example, the β-lactam ring, which is an essential component of many antibiotics such as cephalosporin and penicillin, can be inactivated by β-lactamase enzymes. In addition, enzymes with transferase activity such as acetyltransferase, phosphotransferase, and adenyltransferase can modify antibiotics, resulting in inactivation [45,46]. Another method to become resistant to antibiotics is to modify the target of the antibiotic without affecting cellular functions. For example, the *mecA* gene found in methicillin-resistant *S*. *aureus* (MRSA) encodes PBP2a, a novel penicillin-binding protein that is unaffected by previous β-lactam drugs. Due to this change in the target region, the antibiotic cannot bind as well, leading to resistance [45,47]. As a further resistance mechanism, bacteria can alter the structure of the cell surface to reduce cellular permeability, thereby limiting the amount of antibiotics that can penetrate the cell. In addition, bacteria such as MRSA can become even more resistant to drugs and the host’s immune system through the formation of aggressive biofilms [45,48]. Antibiotics can also activate the bacterial two-component system, also known as two-component signal (TCS), which regulates gene expression in response to external stimuli. Genes involved in antibiotic resistance, such as genes encoding efflux pumps or enzymes that degrade antibiotics, can be overexpressed as a result of this activation [49]. It consists of the response regulator and the sensor kinase, i.e. at least two proteins. In the case of ciprofloxacin, mutations in the genes that produce DNA gyrase or topoisomerase IV, the antibiotic’s targets, can also lead to the development of resistance. Due to these changes, the drug can no longer bind to the intended target site, allowing the bacteria to grow and multiply while the drug is there [49]. These bacteria have developed sophisticated defense mechanisms, such as enzyme production, target alteration, permeability reduction, biofilm formation, and efflux, to resist the lethal effects of antibiotics. In conclusion, these findings contribute to a better understanding of the cellular adaptations and survival mechanisms employed by negative-gram and positive-gram bacteria exposed to ciprofloxacin.

### 4.2. Clustering analysis

GO analysis of cluster genes under ciprofloxacin stress reveals different KEGG pathways for *E*. *faecium* and *S*. *Typhimurium* due to the different physiological adaptations and resistance mechanisms that these bacteria have evolved in response to antibiotic stress. *E*. *faecium*, a gram-positive bacterium, shows the cluster genes associated with transferase, kinase, and FAD in its KEGG pathways. Transferase and kinase are frequently involved in metabolic processes and may contribute to antibiotic resistance by altering the molecules of antibiotics or the targets they act on, thereby reducing drug efficacy [50]. However, GO analysis of cluster genes in the gram-negative bacterium *S*. *Typhimurium* includes pathways such as the two-component system and CAMP (cationic antimicrobial peptides) resistance. CAMP resistance mechanisms often include changes to the bacterial membrane that prevent cationic antimicrobial peptides from penetrating it, which is a common mode of action for some antibiotics [51–53]. To avoid the negative effects of ciprofloxacin, *S*. *Typhimurium* focuses more on membrane protection and regulatory systems than *E*. *faecium*. However, *E*. *faecium* has been shown to adapt to antimicrobial drugs by altering the chemical structure of its lipid membrane and expressing genes that respond to membrane stress [54]. In addition, certain genes and mobile genetic elements contribute to the pathogenicity and antibiotic resistance of *E*. *faecium* [55–57]. Previous research has shown that *IreB* acts as a negative regulator of cephalosporin resistance in *E*. *faecalis* [58]. Gram-positive and gram-negative bacteria differ in their cell wall structure, which also influences their unique KEGG signaling pathways and resistance mechanisms. Due to their unique physiological adaptations and resistance mechanisms, which are determined by structural variations and the selection pressure imposed by the antibiotic environment, *E*. *faecium* and *S*. *Typhimurium* have different KEGG signaling pathways.

### 4.3. Promoter motif analysis

The similarities of the GO results in the hub genes of *S*. *Typhimurium* and *E*. *faecium* suggest common pathways or functions necessary for the survival and basic physiological functions of both bacteria, including peptidoglycan production, glucose transport, and cellular homeostasis. These common motifs may reflect conserved mechanisms between different bacterial species, particularly in terms of cell structure and metabolism. On the other hand, the differences in GO results may reflect the different ecological niches, pathogenic strategies, or evolutionary histories of the two bacteria. For example, motifs related to biofilm formation and biological regulation found only in *E*. *faecium* could be related to its ability to form biofilms, which is a key factor for its persistence and virulence [59–61]. The specific motifs associated with aerobic respiration and alcohol catabolic process may be related to its potential to survive in a variety of environments, including the gastrointestinal tract of humans and other animals [62,63]. The occurrence of a GO motif related to the ABC transporter complex in *E*. *faecium* suggests that this bacterium plays a special role in nutrient absorption or drug resistance [11,64,65]. The differences in molecular activities, such as cation transmembrane transporters and electron carriers, may be indicative of host interactions or environmental adaptations. Overall, GO analysis highlights both similar and different biological features between *E*. *faecium* and *S*. *Typhimurium* that provide insights into their functional capabilities. These results may be useful for further research into the pathogenic mechanisms of these bacteria and may also contribute to the development of targeted interventions or therapies.

## 5. Conclusion

Our study aimed to understand how the mechanisms of drug resistance of *E*. *faecium* and *S*. *Typhimurium* act at the molecular level to survive under the stress of ciprofloxacin. Depending on the structure of the cell wall, Gram-negative bacteria rely on two-component systems to cope with external signals such as antibiotic stress. In contrast, gram-positive bacteria utilise various metabolic and enzymatic adaptations to maintain their cellular integrity under antibiotic stress. Their stress response may involve a wider array of metabolic adjustments and enzyme functions to uphold cellular function and integrity. Understanding these differences is critical for the development of targeted strategies to combat antibiotic resistance in these bacteria, as the key regulatory systems and metabolic pathways they rely on under stress are potential targets for new antimicrobial agents. Both *S*. *Typhimurium* and *E*. *faecium* exhibit increased phosphorylation and metabolic processes, highlighting their common adaptive mechanisms under ciprofloxacin stress. However, the different strategies of efflux pumps in both and phospholipid biosynthesis in *Enterococcus* indicated their unique genetic makeup and evolutionary pathway. The shared GO terms related to peptidoglycan production, glucose transport, and cellular homeostasis suggest basic survival pathways. The divergent GO results are likely due to their different ecological niches, pathogenic approaches, and evolutionary histories. In conclusion, these results contribute to a better understanding of the cellular adaptations and survival mechanisms utilized by negative-gram and positive-gram bacteria exposed to ciprofloxacin. Analyzing these complex adaptations will allow us to develop more effective therapeutic strategies and overcome the difficulties caused by drug-resistant bacteria.

## Supporting information

S1 Table The count of retrieved up- and down-regulated genes of drug-resistant species of *Salmonella Typhimurium* and *Enterococcus faecium*. (DOCX)

S2 Table The topological parameters of the subnetworks of upregulated genes and their interactions in the drug-resistant species of *Salmonella Typhimurium* and *Enterococcus faecium*. (DOCX)

S3 Table The differentially expressed hub genes of the drug-resistant species of *Salmonella Typhimurium* and *Enterococcus faecium*. (DOCX)

S1 File The network of predicted relationships between hub genes and milRNAs in *Salmonella Typhimurium* and *Enterococcus faecium*. (XLSX)
