# Supplementary material for: Comparison of the antibiotic resistance mechanisms in a gram-positive and a gram-negative bacterium by gene networks analysis
Source: PLoS One. 2024 Nov 15;19(11):e0311434. doi: 10.1371/journal.pone.0311434 (PMC11567557; doi:10.1371/journal.pone.0311434)
Supplement: S1 Table — (DOCX) [file pone.0311434.s001.docx]

**S1 Table.** The count of retrieved up- and down-regulated genes of drug-resistant species of *Salmonella Typhimurium* and *Enterococcus faecium*

|  | *S. Typhimurium* | *E. faecium* |
| --- | --- | --- |
| Up-regulated | 25 | 432 |
| Down-regulated | 0 | 457 |
