# Supplementary material for: Comparison of the antibiotic resistance mechanisms in a gram-positive and a gram-negative bacterium by gene networks analysis
Source: PLoS One. 2024 Nov 15;19(11):e0311434. doi: 10.1371/journal.pone.0311434 (PMC11567557; doi:10.1371/journal.pone.0311434)
Supplement: S2 Table — (DOCX) [file pone.0311434.s002.docx]

**S2 Table.** The topological parameters of the subnetworks of upregulated genes and their interactions in the drug-resistant species of *Salmonella Typhimurium* and *Enterococcus faecium*

|  | Nodes | Edges | Network density | Clustering coefficient | The average number of neighbors | Characteristics path lengths |
| --- | --- | --- | --- | --- | --- | --- |
| *S. Typhimurium* | 23 | 104 | 0.206 | 0.223 | 9.043 | 1.644 |
| *E. faecium* | 28 | 237 | 0.313 | 0.374 | 16.929 | 1.404 |
