# Supplementary material for: Comparison of the antibiotic resistance mechanisms in a gram-positive and a gram-negative bacterium by gene networks analysis
Source: PLoS One. 2024 Nov 15;19(11):e0311434. doi: 10.1371/journal.pone.0311434 (PMC11567557; doi:10.1371/journal.pone.0311434)
Supplement: S3 Table — (DOCX) [file pone.0311434.s003.docx]

**S3 Table.** The differentially expressed hub genes of the drug-resistant species of *Salmonella Typhimurium* and *Enterococcus faecium*

|  | Protein or gene ID | Method of hubba nodes | | | | Rank | Gene description | Fold change |
| --- | --- | --- | --- | --- | --- | --- | --- | --- |
|  |  | Degree | DMNC | MNC | MCC |  |  |  |
| *S. Typhimurium* | rcsC |  |  |  |  | 1,1,1,1 | Sensory histidine kinase in two-component regulatory system with RcsB; Component of the Rcs signaling system, which controls transcription of numerous genes. RcsC functions as a membrane- associated protein kinase that phosphorylates RcsD in response to environmental signals. The phosphoryl group is then transferred to the response regulator RcsB. | +4.06 |
|  | narL |  |  |  |  | 1,1 | Response regulator in two-component regulatory system with NarX (or NarQ); Regulates anaerobic respiration and fermentation (LuxR/UhpA family) | +3 |
|  | phoQ |  |  |  |  | 1,1,4 | Sensory kinase protein in two-component regulatory system with PhoP; Member of the two-component regulatory system PhoP/PhoQ which regulates the expression of genes involved in virulence, adaptation to acidic and low Mg(2+) environments and resistance to host defense antimicrobial peptides. Essential for intramacrophage survival of S.typhimurium. In low periplasmic Mg(2+), PhoQ functions as a membrane- associated protein kinase that undergoes autophosphorylation and subsequently transfers the phosphate to PhoP, resulting in the expression of PhoP-activated genes (PAG) | +4.54 |
|  | basR |  |  |  |  | 4,4,4,2 | Response regulator in two-component regulatory system with BasS; Member of the two-component regulatory system BasS/BasR. BasR induces the transcription of the ugd, ais, arnBCADTEF and eptA-basRS loci, all involved in resistance to polymyxin. Represses the transcription of pmrD. Plays a role in the adaptation of the organism to the host environment, in particular to neutrophils, and therefore it plays a role in virulence as well | +3.50 |
|  | phoP |  |  |  |  | 4,4,4,2 | Response regulator in two-component regulatory system with PhoQ; Member of the two-component regulatory system PhoP/PhoQ which regulates the expression of genes involved in virulence, adaptation to acidic and low Mg(2+) environments and resistance to host defense antimicrobial peptides. Essential for intramacrophage survival of S.typhimurium. In low periplasmic Mg(2+), PhoQ phosphorylates PhoP, resulting in the expression of PhoP-activated genes (PAG) and repression of PhoP-repressed genes (PRG). In high periplasmic Mg(2+), PhoQ dephosphorylates phospho-PhoP | +3.32 |
|  | rstB |  |  |  |  | 2 | Sensory histidine kinase in two-component regulatory system with RstA | +5.55 |
|  | envZ |  |  |  |  | 3 | Sensory histidine kinase in two-component regulatory system with OmpR; Member of the two-component regulatory system EnvZ/OmpR involved in osmoregulation (particularly of genes ompF and ompC) as well as other genes (By similarity). EnvZ functions as a membrane- associated protein kinase that phosphorylates OmpR in response to environmental signals; at low osmolarity OmpR activates ompF transcription, while at high osmolarity it represses ompF and activates ompC transcription | +2.98 |
|  | rstA |  |  |  |  | 5 | Similar to E. coli response transcriptional regulatory protein (RstB sensor) | +4.01 |
| *E. faecium* | D920_01853 |  |  |  |  | 1,1,1 | efc: **EFAU004_02486** 1.0e-103 HAD superfamily hydrolase K07025; Psort location: Cytoplasmic | Up-regulated |
|  | D920_01857 |  |  |  |  | 2,2,2 | Acyltransferase; KEGG: efc: **EFAU004_02490** 1.1e-92 acyltransferase K00655 | Up-regulated |
|  | D920_02619 |  |  |  |  | 3,3 | KEGG: efc: **EFAU004_01710** 1.4e-131 ribF; riboflavin biosynthesis protein RibF K11753; Psort location: Cytoplasmic | Up-regulated |
|  | D920_01002 |  |  |  |  | 4,4,3 | Oxidoreductase, short chain dehydrogenase/reductase family protein; KEGG: efc: **EFAU004_00167** 6.6e-93 aldose dehydrogenase K00059; Psort location: Cytoplasmic | Up-regulated |
|  | D920_02133 |  |  |  |  | 4,4 | KEGG: efc: **EFAU004_00900** 9.1e-48 guanylate kinase K00942; Psort location: Cytoplasmic | Up-regulated |
|  | D920_02981 |  |  |  |  | 1,5 | Pyridine nucleotide-disulfide oxidoreductase; KEGG: efc: **EFAU004_02225** 2.7e-144 ferredoxin--NADP reductase K00384; Psort location: Cytoplasmic | Up-regulated |
|  | D920_00703 |  |  |  |  | 2 | Transglycosylase; KEGG: efc: **EFAU004_02016** 0. glycosyl transferase family protein K03693; Psort location: CytoplasmicMembrane | Up-regulated |
|  | D920_02792 |  |  |  |  | 3 | e1-E2 ATPase; KEGG: efc: **EFAU004_01228** 0. cation transporter E1-E2 family ATPase; Psort location: CytoplasmicMembrane | Up-regulated |
|  | D920_02266 |  |  |  |  | 4 | Putative penicillin-binding protein 3; KEGG: efc: **EFAU004_00870** 1.5e-212 penicillin binding protein transpeptidase domain protein; Psort location: CytoplasmicMembrane | Up-regulated |
|  | D920_03079 |  |  |  |  | 5 | KEGG: efc: **EFAU004_00538** 3.2e-155 Putative cysteine desulfurase K04487; Psort location: Cytoplasmic | Up-regulated |
|  | D920_00718 |  |  |  |  | 4 | KEGG: efc: **EFAU004_02003** 5.3e-146 thioredoxin reductase K00384; Psort location: Cytoplasmic | Up-regulated |
